# Confirmatory Virucidal Activity of Ionised Active Water S-100® on the SARS-CoV-2 Virus

**DOI:** 10.1155/2022/5995775

**Published:** 2022-06-17

**Authors:** Nathalie Wurtz, Issam Hasni, Audrey Bancod, Bernard La Scola

**Affiliations:** ^1^Aix Marseille University, IRD, AP-HM, MEPHI, 13005 Marseille, France; ^2^Institut Hospitalo-Universitaire Méditerranée-Infection, 13005 Marseille, France

## Abstract

Ionised active water S-100® has been proposed as an original solution for use in dermocosmetics and for the treatment of wounds such as burns and atopic dermatitis. Among the mechanisms of action that are not completely understood, an antimicrobial activity would appear to be important. In the context of the COVID-19 pandemic, we assessed the inactivating efficacy of this solution on SARS-CoV-2 based on the recommendations of the NF-EN-14476+A2 standard. The tests carried out demonstrated that ionised active water S-100® 40% has a virucidal activity on SARS-CoV-2 which is at least 3.1 log after a contact time of 30 seconds and 3.5 log after two minutes at 20°C under clean conditions. Assays were also performed at 4°C and 37°C, and the results obtained are identical to those obtained at 20°C. This demonstration of the virucidal effect of ionised water against SARS-CoV-2 paves the way for the development of usage as an alternative disinfectant in SARS-CoV-2 control.

## 1. Introduction

The electrolysis of water containing a small amount of salt produces a form of water that is useful and is generally known as “functional electrolysed water.” Different types of functional water can be produced depending on the conditions of electrolysis. Electrolysis occurs in a specially designed reactor which allows the cathodic and anodic solutions to be separated and which produces, respectively, electrolysed alkaline water and electrolysed acidic water [1]. Acidic electrolysed water has a pH of 2–3 and is widely used as a sterilising/disinfecting agent, particularly in the food industry and in the medical and dental fields [2–5]. Alkaline electrolysed water has a pH of 10–13 and is mainly used for cleaning industrial products due to its cleansing and antioxidative effects and is used as drinking water, notably in Japan [6]. Advanced water S-100® is a new specific alkaline water produced by the electrolysis of a natural aqueous solution containing electrolytes using a specific electrolysis cell with a pH of 12 (https://en.adwatis.com/m%C3%A9canismes-d-action). The method for production of this water is presented in the JP2007050400A patent (https://patents.google.com/patent/JP2007050400A/en). Schematically, after a deoxygenation step, the water containing 0.3% of Na, P, K, Si, Cl, Ca, and Mg is electrolysed and the fraction containing negative ions is kept. S-100® water is used in dermocosmetics for its cleaning and protective power. In the medical field, its healing effects on burns have been reported [7,8], as well as on atopic dermatosis complicated by infection [9]. In the dental field, Okajima et al. demonstrated the antibacterial effects of this solution and its effectiveness in dental plaque cleaning [10]. In a recent study, this water demonstrated a bactericidal effect against various Gram-negative bacteria due to its bacterial and bacteriostatic effects [11].

Viral inactivation through low and high pH treatments has been widely used [12], and coronaviruses are sensitive to pH variations [13,14]. In particular, it has been shown that severe acute respiratory syndrome coronavirus (SARS-CoV) is inactivated by alkaline (pH > 12) and acidic (pH < 3) conditions [13]. Over the course of the past year, SARS-CoV-2, the causative agent of coronavirus disease 2019 (COVID-19), has rapidly spread around the world and has been classified as a pandemic by the World Health Organization (WHO); to date, more than 500 million cases have been confirmed and more than six million deaths have been recorded worldwide [15]. Recently, Suzuki et al. demonstrated that active ionised water S-100 was effective on enveloped and nonenveloped viruses and Gram-negative bacteria [11], including SARS-CoV-2 by using a conventional plaque-forming assay. In the context of this pandemic and in view of the alkaline properties of active ionised water S-100®, we evaluated the viricidal activity of this solution in *in vitro* cultures of SARS-CoV-2, based on the recommendations of the EN 14476 + A2: 07–2019 standard [16], by using the 50% tissue culture infectious dose (TCID_50_).

## 2. Materials and Methods

### 2.1. Viral Suspension

The SARS-CoV-2 IHUMI2 strain was isolated from a human nasopharyngeal swab as previously described [17] and used for all tests. The 4-passage strains were grown in VERO E6 (ATCC® CRL-1586™) in the minimum essential medium culture medium (MEM medium, Ref. 21090022, Thermo Fisher Scientific) with 4% foetal calf serum (FCS Ref. 10270106, Thermo Fisher Scientific) and 1% L-glutamine (Ref. 25030024, Thermo Fisher Scientific), without antibiotics at 37°C under 5% CO_2_ for 72 hours. Before use, the SARS-CoV-2 strain was filtered on a 0.2*μ* filter and diluted from 10^−1^ to 10^−10^ in an ice-cold MEM medium containing 2% FCS (conservation medium). All experiments involving SARS-CoV-2 cultures were carried out in a biosafety level 3 laboratory and conducted under appropriate conditions. The experiments were performed in triplicate.

### 2.2. Cell Line

The VERO-81 cell lines (ATCC® CCL-81™) were inoculated in 96-well flat-bottom microplates (Ref. 020035, Dutscher) at 2 ∗ 10^5^ cells/ml, without antibiotics and incubated to reach confluence at 37°C under 5% CO_2_.

### 2.3. Viral Titration

The product test solution was replaced with hard water. Hard water was used throughout the test as distilled water would have deleterious effects on the virus. The pH of the hard water is adjusted to 7, and 9.7 ml of hard water was added instead of the product test solution. 100 *μ*l of each virus dilution was transferred into six wells containing pre-established VERO-81 cells, beginning with the highest dilution. After one hour of incubation at 37°C in the presence of 5% of CO_2_, 100 *μ*l of the complete growth medium (MEM medium + 10% FCS) was added to each well. The cytopathogenic effects (CPEs) were read with an inverted microscope after five to seven days of incubation. The calculation of the viral titre was determined using the Spearman–Kärber method, as previously described (log TCID_50_/ml) [18].

### 2.4. Solution to Be Tested

Ionised active water S-100® 40% (Adwatis, La Chaux-de-Fonds, Switzerland) was evaluated for its virucidal activity against SARS-CoV-2. Throughout this article, this water will be called “ionised water” or “this solution.”

### 2.5. Cytotoxicity

To detect any possible morphological alteration of the cells by the product to be tested, 9.7 ml of ionised water (40%) was mixed with 200 *μ*l of bovine serum albumin (BSA) (final concentration at 0.3 g/L) and 100 *μ*l of hard water. Serial ten-fold dilutions were performed in an ice-cold storage medium (from 10 to 1 to 10–5), and 100 *μ*l of each dilution was inoculated into six wells containing pre-established VERO-81 cells in a monolayer, starting with the highest dilution. After one hour of incubation at 37°C in the presence of 5% CO_2_, 100 *μ*l of the complete growth medium was added to each well. The cytotoxic effect was assessed using an inverted microscope after five to seven days of incubation at 37°C in the presence of 5% CO_2_.

### 2.6. Interference Control: Control of Cell Sensitivity

The interference control was used to verify that the sensitivity of cells to viral infections was not affected by the treatment with the solution to be tested. Comparative viral titre assays were performed on cells treated or not treated with the solution to be tested. 100 *μ*l of the lowest apparently noncytotoxic dilution of ionised water (40%) (no microscopic cell deterioration) which corresponds to dilution 10^−1^, or 100 *μ*l of hard water was distributed in six wells (by dilution) to which 100 *μ*l of the complete culture medium was added. After one hour at 37°C with 5% CO_2_, the supernatant was removed and 100 *μ*l of the different virus dilutions from 10 to 1 to 10–10 (diluted previously in storage medium) was deposited on the treated or untreated cells. The virus was titrated as previously described.

### 2.7. Validation of the Effectiveness of Stopping the Activity of the Product

For elimination of the virucidal activity of ionised water (40%), the cold dilution technique was used. 9.7 ml of the solution was mixed with 200 *μ*l of an interfering substance at 0.3 g/L and 100 *μ*l of the storage medium. 500 *μ*l of this mixture was added to 4 ml of the cold storage medium and 500 *μ*l of viral suspension. The mixture was incubated in the melting ice bath for 30 minutes. After incubation, a series of 10-fold dilutions (from 10 to 1 to 10–10) were prepared, and the virus was titrated as previously described.

### 2.8. Inactivation Control of the Virus with 0.7% Formaldehyde

The suspension of the virus was subjected to virucidal tests against 0.7% formaldehyde solution as a reference substance for viral inactivation in order to control the uniformity of the behaviour of the virus stock to the chemical agents during the time. 2 ml of a viral suspension was mixed with 8 ml of PBS and 10 ml of formaldehyde 1.4%. After 5, 15, and 30 minutes of contact time, 200 *μ*l of the mixture was added to a tube containing 1.8 ml of the ice-cold storage medium, followed by an additional 10-fold dilution. The mixture was left in a melting ice bath. Dilutions up to 10–8 were prepared in an ice-cold storage medium, and the virus was titrated as previously described.

### 2.9. Determination of Virucidal Activity

To test the virucidal activity of the product, 9.7 ml of ionised water (40%) was mixed with 200 *μ*l of an interfering substance and 100 *μ*l of a viral suspension. After 30 seconds and two minutes of contact time at 4°C, 20°C, or 37°C, 500 *μ*l was mixed with 4.5 ml of the storage ice-cold medium and placed in a melting ice bath. Within 30 minutes, 10-fold dilutions were performed in the ice-cold storage medium until dilution 10–8 and the virus was titrated as previously described.

### 2.10. Statistical Analysis

Statistical analyses were performed in the statistical environment *R* v.4.0.5. A *t*-test was applied on log10 transformed TCID50/ml values to compare the mean of SARS-CoV-2 titres between control (hard water 97%) and ionised water 40% after 30 seconds and 2 min exposure at 4°C, 20°C, and 37°C. A second analysis was performed to compare the mean of SARS-CoV-2 titres between ionised water 40% after 30 seconds and 2 min exposure at 4°C, 20°C, and 37°C and ionised water 40% after 30 seconds and 2 min exposure at 4°C, 20°C, and 37°C. Groups of samples are considered to be statistically different if the *p* value is less than 0.05.

## 3. Results and Discussion

### 3.1. Results

#### 3.1.1. Cytotoxicity

The results of the cell cytotoxicity of ionised water (40%), formaldehyde 0.7%, and hard water are presented in Table 1. No cytotoxicity on VERO-81 cells was observed from dilution 10^−1^ to 10^−5^ of ionised water (40%) and hard water (control). Low toxicity on VERO-81 was observed up to the dilution 10–3 with formaldehyde 0.7%.

#### 3.1.2. Titration of SARS-CoV-2 by Cytopathic Effects

The average SARS-CoV-2 TCID_50_ obtained for the tests was 6.9 log ± 0.2.

#### 3.1.3. Interference Control: Control of Cell Sensitivity

Comparative titrations of the virus were carried out on the VERO-81 cells previously treated with ionised water 40% or not treated. In parallel, formaldehyde 0.7% and hard water were used as controls. The results are presented in Table 2.

The ionised water 40% tested did not show any appreciable influence on the method of titration of the SARS-CoV-2 virus, as the difference of the titre compared to the initial control of TCID_50_ of SARS-CoV-2 was less than 1 log. The same results were obtained for formaldehyde 0.7%, as the difference compared to the control was less than 1 log. Hard water had no impact.

#### 3.1.4. Validation of the Effectiveness of Stopping the Activity of the Product

Comparative titrations of the virus were carried out after the ionised water 40% activity was stopped by ice-cold dilution and incubation. The average TCID_50_ of SARS-CoV-2 was 6.7 ± 0.3. The inactivation method by ice-cold dilution is validated since the difference in the titre compared to the initial TCID_50_ was less than 0.5 log (difference 0.2 log).

#### 3.1.5. Inactivation Control of the Virus with Formaldehyde (0.7%)

In parallel to the assay, a control using formaldehyde (0.7%) was used. Formaldehyde was put in contact with the SARS-CoV-2 virus for 5, 15, and 30 minutes and TCID_50_ was determined. The results are presented in Table 3. The difference in TCID_50_ of the SARS-CoV-2 virus in the control assay compared to the initial titre is between 2.7 and at least 4.9 log for 5 minutes, 15 minutes, and 30 minutes of the contact time.

#### 3.1.6. Determination of the Virucidal Activity of Ionised Water

Comparative titrations of the SARS-CoV-2 virus were carried out after treatment of the virus with ionised water 40% for 30 seconds and two minutes at 3 different temperatures, 4°C, 20°C, and 37°C. Two controls using hard water were performed alongside the assay. The results are presented in Table 4. The tests carried out demonstrated that the ionised water 40% used at 97% has a virucidal activity on SARS-CoV-2 at least 3.1 log and 3.5 log, after a contact time of 30 seconds and two minutes, respectively, at 20°C under clean conditions, at least 2.5 log and 3.0 log, after a contact time of 30 seconds and two minutes, respectively, at 4°C under clean conditions, and at least 3.2 log and 3.5 log, after a contact time of 30 seconds and two minutes, respectively, at 37°C under clean conditions.

**Figure 1:**
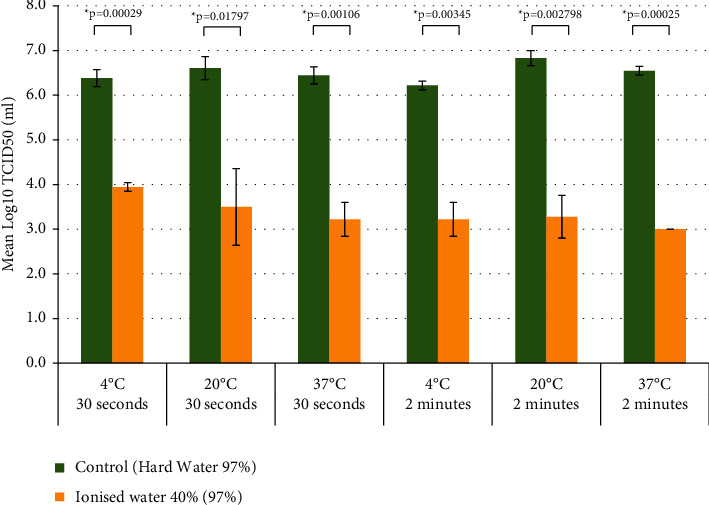
Comparison of the mean TCID50/ml obtained for SARS-CoV-2 infected cells in the control (hard water 97%) and ionised water 40% (97%) after 30 seconds and 2 min exposure at 4°C, 20°C, and 37°C.

The difference in titration of SARS-CoV-2 observed between the virus treated with the ionised water 40% and control (hard water) is statistically significant after 30 seconds of exposure with a *p* value = 0.01797 and after 2 minutes of exposure with a *p* value = 0.002798 at 20°C, after 30 seconds of exposure with a *p* value = 0.00029 and after 2 minutes of exposure with a *p* value = 0.00345 at 4°C, and after 30 seconds of exposure with a *p* value = 0.00106 and after 2 minutes of exposure with a *p* value = 0.00025 at 4°C. These results are presented in Figure 1.

The difference in titration of SARS-CoV-2 observed between the virus treated with the ionised water 40% at 4°C, 37°C, and 20°C during 30 seconds or 2 minutes of the contact time is not statistically significant (*p* > 0.05).

### 3.2. Discussion

The virucidal activity tests of the ionised water were carried out based on the recommendations of the EN 14476 + A2: 07–2019 standard [16]. A test is only valid if several criteria are met. First, the cytotoxicity of the test solution must not affect cell morphology and growth. We showed that the ionised water (40%) did not affect or modify VERO-81 cells. Then, comparative titrations of the virus on cell cultures pretreated with the product result in a difference of <1 log in the viral titre. We showed that VERO-81 cells previously treated with ionised water 40% did not significantly modify the viral titre as the difference of TCID_50_ was 0.3. When checking the effectiveness of stopping the activity of the product, the difference in the viral titre with the test suspension should be less than 0.5 log. We showed that ice-cold dilution of the product is effective to stop the activity of ionised water 40%, as the difference in the titre compared to the initial TCID_50_ is less than 0.5 log (difference 0.2 log).

Nevertheless, the standard gave a reduction of titre that should be observed with formaldehyde for different contact times and several viruses, namely, poliovirus, adenovirus, murine norovirus, parvovirus, and vaccinia virus. No reference is currently provided for the SARS-CoV-2 virus. On the other hand, we encountered difficulties in interpreting the results with formaldehyde as this agent is a known chemical fixative that blocks the growth of cells. It would therefore be interesting to use another reference test.

The last point which is problematic is the titre of the viral suspension. The standard recommends using at least 10^8^ TCID_50_/ml in order to be able to show a titre reduction of at least 4 log in the test. Unfortunately, we did not manage to get such a high titre of SARS-CoV-2 virus. The maximum TCID_50_/ml that we obtained was 10^7^ TCID_50_/ml. Several tests were performed to try to increase viral titre, namely, increasing the number of VERO-81 cells, reducing or increasing the time of preincubation of the virus before the assay (24h–48 h to 72h–96 h), and reducing the quantity of the initial virus inoculated. None of these tests made it possible to increase the SARS-CoV-2 viral titre. Nevertheless, the assays using the ionised water were performed, and we could conclude that this solution has a virucidal activity at least 3.1 log for a contact time of 30 seconds and 3.5 log for a contact time of two minutes at 20°C, at least 2.4 log for a contact time of 30 seconds and 3.00 log for a contact time of two minutes at 4°C, and at least 3.2 log for a contact time of 30 seconds and 3.5 log for a contact time of two minutes at 37°C against SARS-CoV-2. No statistical differences were observed at 4°C, 20°C, or 37°C, suggesting that the ionised water can be used in both winter and summer. Our results are consistent with a previous work, testing the effect of this active water on SARS-CoV-2 by using a conventional plaque-forming assay [16].

The mechanisms of action of this water are little known and partially hypothetical. First, ionised water has a confirmed pH of 12 and this low pH is supposed to be the main mechanism of a microbicidal effect. In two recent studies, active water showed a bactericidal effect against various Gram-negative bacteria, as well as enveloped and nonenveloped viruses, including SARS-CoV-2 [11]. Two studies showed that exposure of coronaviruses (SARS-CoV and canine coronavirus) to extreme basic or acidic conditions caused inactivation of the virus, while the virus remained stable within a range of neutral pH [13,14]. Several studies suggested that spike glycoprotein of coronavirus, which regulates biological functions, such as attachment to cells, fusion of the viral envelope with host cell membranes, and cell to cell fusion, might be sensitive to pH variations probably by changing the infectious nature of viral particles [19–21]. But besides the effect of an extremely low pH that is probably sufficient to kill nearly all microorganisms, other mechanisms have been proposed such as the “double electric layer” theory. Active water contains novel molecular structures with negative ionic charges. Once in contact with any kind of the surface, skin, mucosa, material, or particle, water ions interact by reorganizing electric charges. The surface remains polarized and actively prevents any future building up of foreign particles. If the impact of this effect is probably negligible in the case of bacteria, it has certainly an effect on SARS-CoV-2 infection of susceptible cells by introducing alteration of electrostatic forces between SRAS-CoV-2 spike that contains receptor binding domains (RBDs) and the angiotensin-converting enzyme (ACE) 2 receptor. Indeed, SARS-CoV-2 has been shown to have a higher electric field line density than that of SARS-CoV that leads to better attachment to a receptor and higher transmissibility as compared to SARS-CoV [22], in example through a positive-charged patch on RBD contributed by Lys417 [23].

In the COVID-19 epidemic, high levels of SARS-CoV-2 are detected in the upper respiratory tract of symptomatic and asymptomatic individuals and the virus spread quickly among people, primarily through droplets of saliva discharged from the nose when an infected person coughs, sneezes, talks, or sings [24]. In the context of the health crisis, it has been suggested that a nasal wash may be beneficial in upper respiratory infectious diseases, such as rhinoviruses, influenza viruses, or SARS-CoV-2 [25–27]. However, several otolaryngologist societies recommend limiting nasal lavages, believing that this may be associated with viral spread to the lower airways [28]. In contrast, however, Ramalingam et al. have shown that hypertonic saline nasal irrigation and gargling reduced the duration of coronavirus upper respiratory tract infection by an average of two and a half-day [29]. Furthermore, work using another product with a local antiseptic activity, povidone-iodine, has shown efficacy in preventing contamination by SARS-CoV-2 by using the product in nasal spray and/or gargle [30,31]. So, ionised water 40% may be effective in reducing the amount of coronavirus present in individuals' noses, potentially reducing the risk of infection by the virus that causes COVID-19. Currently, there is no evidence that a nasal spray could prevent SARS-CoV-2 transmission; thus, to validate this hypothesis, a clinical trial is required.

## 4. Conclusions

Finally, indirect contact transmissions of SARS-CoV-2 involving contact with contaminated surfaces are also possible [32]. Several studies have shown that SARS-CoV-2 can remain viable on surfaces, particularly plastic and stainless steel [33,34], meaning that effective disinfectants can prevent indirect contact transmission. Therefore, contaminated surfaces and solutions are a reservoir for transmission through fomites, meaning that effective hygiene and environmental decontamination are crucial to preventing the spread of COVID-19 [35–38]. Previous studies have shown that heat, chemical inactivating agents, UV light, gamma radiation, and a variety of detergents are effective at inactivating SARS-CoV-1 and MERS-CoV [39,40], as well as SARS-CoV-2 [41,42]. It can be concluded that the ionised water (40%) could potentially significantly reduce infection by the virus and would be an easy and safe option, in combination with other precautionary measures already in place, to limit transmission of the SARS-CoV-2 within the population at large.

## Figures and Tables

**Table 1:** Cytotoxicity of products on VERO-81 cells.

Product tested	Concentration (%)	Dilutions^a^				
		10–1	10–2	10–3	10–4	10–5
Ionised water (40%)	97	0/6	0/6	0/6	0/6	0/6
Formaldehyde	0.7	6/0	6/0	5/1	0/6	0/6
Hard water	98	0/6	0/6	0/6	0/6	0/6

^a^The first number represents the number of wells where the cells were damaged by the treatment. The second number represents the number of wells where the cells were normal.

**Table 2:** Comparative titrations of the SARS-CoV-2 virus after treatment of VERO-81 cells with ionised water 40%, formaldehyde (0.7%), and hard water.

Product tested	Dilution	Mean log10 TCID_50_/ml	Reduction titre
Ionised water (40%)	10^–1^	6.6 ± 0.1	0.3
Formaldehyde	10^–4^	6.6 ± 0.5	0.3
Hard water	10^–1^	6.7 ± 0.3	0.2

**Table 3:** Comparative titrations of the SARS-CoV-2 virus after treatment with formaldehyde (0.7%) after 5, 15, and 30 minutes of the contact time.

Product tested	Concentration (%)	Contact time	Mean log10 TCID_50_/ml	Reduction titre
Formaldehyde	0.7	5 minutes	4.2 ± 0.9	2.7
Formaldehyde	0.7	15 minutes	3.6 ± 0.9	3.3
Formaldehyde	0.7	30 minutes	<2.0	>4.9

**Table 4:** Comparative titrations of the SARS-CoV-2 virus after treatment with the ionised water 40% after 30 seconds and two minutes of the contact time at 4°C, 20°C, and 37°C.

Product tested	Concentration (%)	Temperature (°C)	Contact time	Mean log10 TCID_50_/ml	Mean minimal reduction titre
Ionised water (40%)	97	20	30 seconds	<3.5 ± 0.9	3.1
Hard water	97	20	30 seconds	6.6 ± 0.3	—
Ionised water (40%)	97	20	2 minutes	<3.3 ± 0.5	3.5
Hard water	97	20	2 minutes	6.8 ± 0.2	—
Ionised water (40%)	97	4	30 seconds	3.9 ± 0.1	2.5
Hard water	97	4	30 seconds	6.4 ± 0.2	—
Ionised water (40%)	97	4	2 minutes	3.2 ± 0.2	3.0
Hard water	97	4	2 minutes	6.2 ± 0.4	—
Ionised water (40%)	97	37	30 seconds	3.2 ± 0.4	3.2
Hard water	97	37	30 seconds	6.4 ± 0.2	—
Ionised water (40%)	97	37	2 minutes	3 ± 0.0	3.5
Hard water	97	37	2 minutes	6.5 ± 0.1	—

## Data Availability

The data used to support the findings of this study are included within the article.
